# Mental stress and physical activity interact with the genetic risk scores of the genetic variants related to sweetness preference in high sucrose‐containing food and glucose tolerance

**DOI:** 10.1002/fsn3.1632

**Published:** 2020-05-21

**Authors:** Sunmin Park, Meiling Liu, Mi Young Song

**Affiliations:** ^1^ Department of Food and Nutrition Obesity/Diabetes Research Center Hoseo University Asan South Korea; ^2^ Department of Food Science and Nutrition Woo Song University Daejeon South Korea

**Keywords:** genetic variants, glucose tolerance, sucrose intake, sweet taste, waist circumference

## Abstract

We hypothesized that subjects with genetic variants that increase sweet taste preference would consume more sucrose‐containing foods and have altered energy and glucose metabolisms, which would have interactions with lifestyles. Korean genome and epidemiology study (KoGES) was conducted to determine genetic variants and lifestyles including nutrient intakes by the Korean Center for Disease and Control during 2004–2013. Subjects were 8,842 adults aged 40–69 years in Ansan/Ansung cohorts in Korea. The associations between genetic risk scores(GRS) selected for influencing higher sweet preference and energy and glucose metabolism were examined using logistic regression after adjusting for covariates. GRS included 8 SNPs, *TAS1R2*_rs61761364, *SLC2A5*_rs11121306, *SLC2A7*_ rs769902, *SLC2A5*_rs765618, *TRPM5*_rs1965606, *TRPV1*_rs224495, *TRPV1*_ rs8065080, and *TRPV1*_rs8078502. Sweet taste preference was higher by 1.30‐folds in high GRS than in low GRS (*p* < .0001). Consistent with sweet taste preference, carriers with high GRS had a higher intake of sucrose‐containing foods by 1.25 (1.08–1.46)‐fold than those with low GRS after adjusting age, gender, BMI, and energy intake. However, glucose intolerance risk was rather lower by 0.861 (0.76–0.98)‐fold in high GRS than low GRS (*p* < .05). GRS tended to interact with mental stress to affect sucrose intake (*p* = .048). Only in low mental stress levels, sucrose‐containing food intake was higher in high GRS than low GRS. There was an interaction of GRS with physical activity to influence glucose intolerance. Serum glucose concentrations were lower by 0.808‐folds in high GRS than low GRS only in a high physical activity state. In conclusion, adults with genetically high sweet taste preference had a positive association with high sucrose‐containing food intakes and improved glucose tolerance. The genetic impact on sweetness preference was associated with offset by high mental stress and lack of physical activity.

## Graphical abstract

Eight genetic variants associated with sweet taste preference were selected in the genes involved in taste receptor signaling. Adults carrying genetically high sweet taste preferences were associated with consuming higher sucrose‐containing food intakes and improving glucose tolerance. High mental stress and lack of physical activity quenched the genetic impact.

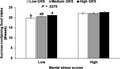

## 1 INTRODUCTION

Sucrose intake especially sucrose‐sweetened beverage contribute to excess energy intake and increased glycemic load by providing energy source with increased insulin secretion (Schlesinger et al., 2017). Various non‐nutritive sweeteners have been substituted for sucrose and have no or few calorie and glycemic index. However, non‐nutritive sweeteners are involved in stimulating glucose transport, incretin and insulin secretion, and effects on glucose tolerance (Chan, Hashemi, & Subhan, 2017). Moreover, non‐nutritive sweeteners are reported to elicit metabolic changes that contribute to obesity and glucose intolerance in a cell‐based, animal model, and human studies, although the results are inconsistent (Pearlman, Obert, & Casey, 2017; Suez et al., 2014). Non‐nutritive sweeteners mediate activation of sweet taste receptors in oral, intestine, pancreatic β cells and brain, and the modulation of the gut microbiome (Rother, Conway, & Sylvetsky, 2018; Suez et al., 2014). The results have suggested that the activation of taste receptor and/or sweet taste preference may influence energy and glucose metabolism. Therefore, genetic differences in taste receptors may be differently influenced by sucrose‐containing foods and genetic variants of taste receptors affect the intake of sweet foods.

Two types of taste receptors (*TAS1R* and *TAS2R*) in the tongue and gastrointestinal cells are integral plasma membrane proteins that recognize substances, received taste information from these substances, and deliver the information to the intracellular messenger, α‐gustducin (Lee & Owyang, 2017). The signal stimulates phospholipase C‐β2 and inositol trisphosphate to release intracellular Ca^++^ and activates transient receptor potential cation channel (*TRP*) M5 (*TRPM5*). Glucose transporters such as *SLC2A7* (GLUT7) and *SLC2A5* (GLUT5) are involved in the signal processes, and they are involved in sweet taste and sweet preference (Merigo, Benati, Cristofoletti, Osculati, & Sbarbati, 2011; Robino et al., 2015). These signals activate adjacent sensory afferent neurons that deliver the signals by vagus nerve to brain centers where taste perception occurs (Lee & Owyang, 2017). *TAS1R* identifies sweet matter and *TAS2R* recognizes bitter compounds including toxins, acids, and alkaloids. Taste receptors may be involved in sour, salty, and umami taste. Spicy hot taste is detected by transient receptor potential cation channel subfamily V member 1 (*TRPV1*) as pain, and *TRPV1* is activated by noxious heat, capsaicin, and ethanol (Allen, McGeary, & Hayes, 2014). *TRPV1* knocked out mice have a higher preference for ethanol and consume more ethanol than wild‐type mice (Blednov & Harris, 2009). Taste perception such as sourness, sweetness, saltiness, and bitterness are influenced by each other. For example, a small amount of salt in the food increases the sweet taste, indicating that taste receptors interact with different taste compounds. Thus, taste recognition‐related genes may interact with preferences for sweet tastes. Genes not directly related to sweet taste like *TRPV1* can interact with sweet preference.

Taste receptors influence not only taste preference but also glucose metabolism. *TAS1R3* knockout mice exhibit the abolishment of taste preferences for sucrose solutions and reduced insulin sensitivity and glucose tolerance (Murovets, Bachmanov, & Zolotarev, 2015). After intraperitoneal, but not intragastric, administration of glucose exacerbates glucose intolerance, indicating glucose homeostasis is influenced by *TAS1R3* in tissues (brain and pancreatic β cells) other than the gastrointestinal tract (Murovets et al., 2015). The activation of taste receptors has an association with glucose metabolism involved in insulin secretion (Murovets et al., 2015). The modulation of glucose metabolism by the loss of *TAS1R3* may be associated with the activation of taste receptors in the gut, brain, and pancreatic β cells. We hypothesized that people with genetic variants related to sweet taste preference may consume more sucrose‐containing foods and exhibit the perturbation in energy, lipid, and glucose metabolism and that those effects may interact with other lifestyle factors. We examined the hypothesis in 8,842 Korean middle‐aged adults.

## 2 MATERIALS AND METHODS

### 2.1 Participants

The data used in this study were collected in 2001 from subjects of the Ansan/Ansung cohort of the KoGES (Hong & Oh, 2012). Briefly, the participants were recruited from two community‐based epidemiological cohorts: the urban and rural communities of Ansan and Ansung city, respectively. A total of 8,842 subjects (4,183 men and 4,659 women; age, 40–69 years) were recruited. This study was approved by the institutional review board of the Korean National Institute of Health for the KoGES and Hoseo University. Written informed consent was received from each participant.

### 2.2 Basic characteristics and biochemical measurement

All participants had resided within the survey area for at least 6 months, and they were mentally healthy without serious diseases such as cancers. Anthropometric and biochemical examinations of the cohorts were conducted in 2001. Information on age, education, income, smoking history, alcohol consumption, dietary consumption, and physical activity was collected during a health interview.

Height, weight, waist circumference, body fat, and blood pressure were measured. Body mass index (BMI) was calculated by weight (kg)/square of height (m^2^). Blood was collected after 10 or more h of fasting and separated by centrifugation. Serum glucose and insulin levels were measured using an AutoAnalyzer (Hitachi 7600; Hitachi) and an ELISA kit, respectively.

Smoking status was divided into three categories: current smoker, past smoker, and never‐smoker. Alcohol consumption was assessed by asking questions about the participants' drinking behaviors during the last month before the interview. Alcohol consumption status was categorized into two groups according to average daily alcohol consumption: nondrinker and light drinker (<20 g), moderate and heavy drinker (≥20 g). The physical activity level was divided by light, moderate, and heavy activity that was scored by 1, 2, and 3. Daily total physical activity was calculated by the summation of multiplying each activity level by time and the scores indicated the hours of reference (light) activity per day. The cut‐point scores for physical activity were 10. Mental stress was evaluated by 6 questionnaires about mental stress at home and work. Each question was scored into 0 (no stress), moderate stress (a), and high stress (b). Mental stress was calculated with a summation of scores in each item and the summed score of mental stress was divided into 6 items in the workplace and family life. Lowest and highest scores of mental stress were 0 to 12. The cut‐point for high mental stress was ≥10 of the score. Preferences for each taste like sweet, salty, spicy, sour, and oily foods were asked as different questions: “Do you like sweet foods?”, “Do you like salty foods?” and so on. Each question was answered as one of the following answers: “very dislike”, “dislike”, “moderate”, “like”, or “very like” (Matsushita et al., 2009; Shin, Lee, & Kim, 2018). Preferences for each taste were categorized into two groups as “low preference”, including “very dislike” and “dislike”, and as “high preference”, including the rest of the scale for each taste.

### 2.3 Definition of metabolic syndrome and type 2 diabetes

As based on the American Heart Association definition, metabolic syndrome was defined as a cluster having 3 or more of the following: (a) abdominal obesity (waist circumference ≥90 cm for men and ≥85 cm for women); (b) elevated blood pressure (average systolic blood pressure ≥130 mmHg or diastolic blood pressure ≥85 mmHg) or current blood pressure medication use; (c) low HDL cholesterol level (<40 mg/dl for men and <50 mg/dl for women); (d) elevated serum triglyceride level (≥150 mg/dl) or current antidyslipidemic medication use; and (e) elevated fasting blood glucose level (≥110 mg/dl) or current antidiabetic medication use (Park, Ham, & Lee, 2015; Yamagishi & Iso, 2017). Participants who were taking medication for dyslipidemia, hyperglycemia, and hypertension were included in the metabolic syndrome group.

Type 2 diabetes was defined as fasting serum glucose levels ≥126 mg/dl or 2‐hr serum glucose levels ≥200 mg/dl during an oral glucose tolerance test (OGTT) or current use of antidiabetic medications, whereas glucose intolerance was categorized as 100 mg/dl <fasting serum glucose levels <126 or 140 mg/dl <serum glucose levels 120 min < 200 mg/dl after oral glucose loading (Pippitt, Li, & Gurgle, 2016). Insulin resistance was determined using the homeostasis model assessment (HOMA) estimate of insulin resistance [HOMA‐IR = fasting insulin (µIU/ml) × fasting glucose (mM)/22.5] (Kim, Kim, Daily, & Park, 2018). Insulin secretion capacity was represented by HOMA‐β, and calculated as (20 × fasting insulin)/(fasting glucose − 3.5).

### 2.4 Assessment of foods and nutrient intake

The Korean dish‐based semi‐quantitative food frequency questionnaire (SQFFQ) was used to assess the long‐term food intake of the 8,830 participants in the KARE studies. The validity and reproducibility of this SQFFQ were evaluated by previous studies in the Korean population (Ahn et al., 2007). This SQFFQ demonstrated moderate correspondence with the four 3‐day food records, taken during all 4 seasons to make them to 12‐day food records, in the previous study (Ahn et al., 2007). This questionnaire requested information regarding the participant's average consumption of food items during the last l year. The SQFFQ included 103 food items and the intake of food frequencies was divided into nine categories: never or seldom, once a month, two to three times a month, one to two times a week, three to four times a week, five to six times a week, once a day, twice a day, and three times or more every day. The amount of food intake at once was checked as "more," "equal," or "less" based on the portion size. SQFFQ data were converted into food intake per day in each food category by multiplying the number of times each food was consumed by the amount of food intake. The portion size was given in each food category and participants selected the frequencies based on the defined portion size. The daily intake was computed based on the midpoint of the reported frequency category for each food item. For example, when one food item was checked as 2–4/week, and it was calculated to be 3/7 or 0.43 times/day.

Sucrose‐containing food intake was calculated into 2 categories (a) by summing the servings of the foods (coffee with sucrose, soda, and candy) mainly containing sucrose for 1 week and (b) by summing the servings of foods (coffee with sucrose, soda including coke, candy, cake, snack, pastry, and ice cream) for sweet taste for 1 week. Daily nutrient intake was calculated from the semi‐quantitative food frequency questionnaires. From the food intake, energy and nutrients such as protein, carbohydrates, fat, fiber, total vitamin A, vitamin C, Na, Ca, and K were calculated using the Can‐Pro 2.0 nutrient intake assessment software developed by the Korean Nutrition Society. Daily estimated energy requirement and recommended nutrient intakes were obtained from the Korean dietary reference intake according to age and gender (Paik, 2008).

### 2.5 Genotyping, imputation of genotypes, and quality control

The genotype data were graciously provided by the Center for Genome Science, Korea National Institute of Health. Most DNA samples were isolated from the peripheral blood of participants and genotyped using the Affymetrix Genome‐Wide Human SNP array 5.0 (Affymetrix) (Hong & Oh, 2012).

Imputation of genotypes was carried out with the IMPUTE (v2.644) containing the 1,000 Genomes phase I integrated variant call set release (version 3) in NCBI build 37 (hg19) as a reference panel. High imputation quality was included (proper info >0.5) (Hwang et al., 2016; Kobayashi et al., 2010).

The accuracy of the genotyping was examined using the Bayesian Robust Linear Modeling with Mahalanobis Distance genotyping algorithm (Rabbee & Speed, 2006). Samples with low genotyping accuracies of < 98%, high missing genotype call rates (≥ 4%), high heterozygosity (>30%), or gender biases were excluded. SNPs were met Hardy–Weinberg equilibrium (HWE) *p* < .05, minor allele frequency (MAF) <.01, and SNP missing rate >.1. Linkage disequilibrium analysis between the genetic variants included in the genetic risk score (GRS) was performed using locus zoom. Haplotype associations were carried out in GPLINK v1.07 by linear regression.

### 2.6 SNP selection for sweet taste preference and calculation of GRS

Genome‐wide association study (GWAS) was conducted to explore the genetic variants for sweet taste perception after adjusting for age, gender, and residence area using 1,000 Genomes imputed SNPs (Kobayashi et al., 2010; Lee & Owyang, 2017). Among the selected genetic variants to influence taste perception and its signaling, the genetic variants with lower *p*‐value and meeting HWE criteria were selected. Genetic variants from *AS1R1*, *TAS1R2*, *TAS1R3*, *TAS2R*, *TRP*, *TRPM5*, *TRPV1*, *SLC5A1*, *SLC2A2*, *SLC2A5*, *SLC5A4*, and acid‐sensing ion channel subunit 1 (*ASIC*) were selected. Among the genetic variants of the selected genes, genetic variants with *p*‐value <.05 were selected for calculating the genetic risk score (GRS).

GRS was calculated by summation of the number of risk alleles (“like more sweet taste”) from each selected SNP without weighting by beta coefficients. Nonrisk, heterozygotes, and risk alleles were given a score of 0, 1, and 2, respectively. As a result, high GRS indicated high sweet taste preference calculated from self‐reported sweet taste score. The GRSs were divided into 3 categories (0–4, 5–7, and > 7) by its tertiles (low GRS, Medium‐GRS, high GRS) indicating that the group with lower values included the persons with fewer risk alleles of the genetic variants.

### 2.7 Statistical analysis

Statistical analyses were performed using GPLINK version 2.0 (http://pngu.mgh.harvard.edu/~purcell/plink) and SAS (version 9.3; SAS Institute). The descriptive statistics of participants for categorical variables, such as gender, taste perception, and dietary habits, were obtained by determining frequency distributions. Frequency distributions by classification variables were analyzed using the chi‐squared test. The descriptive statistics of continuous variables are expressed as means with standard deviations (SDs). Multivariate adjustments for comparisons of continuous variables were carried out by generalized linear models. The results were adjusted for age, gender, and residence area. Adjusted odds ratios (ORs) and 95% confidence intervals (CIs) of GRSs were calculated for the taste perception and risk of type 2 diabetes while controlling for covariates. To examine the interaction between the GRS and sucrose intake and lifestyles, separate multivariate regression models were used for including the corresponding main effects and interaction terms in addition to the potential confounders. Next, ORs and 95% CIs were calculated for the taste perception and type 2 diabetes risk according to sucrose intake and lifestyles while controlling for covariates using the multivariable logistic regression method. The confounders used for the analysis were age, gender, residence area, BMI, daily energy intake, physical activity, alcohol intake, smoking status, mental stress, and carbohydrate and fat intake as indicated.

## 3 RESULTS

### 3.1 Characteristic of genetic variants to be associated with taste perception

**FIGURE 1:**
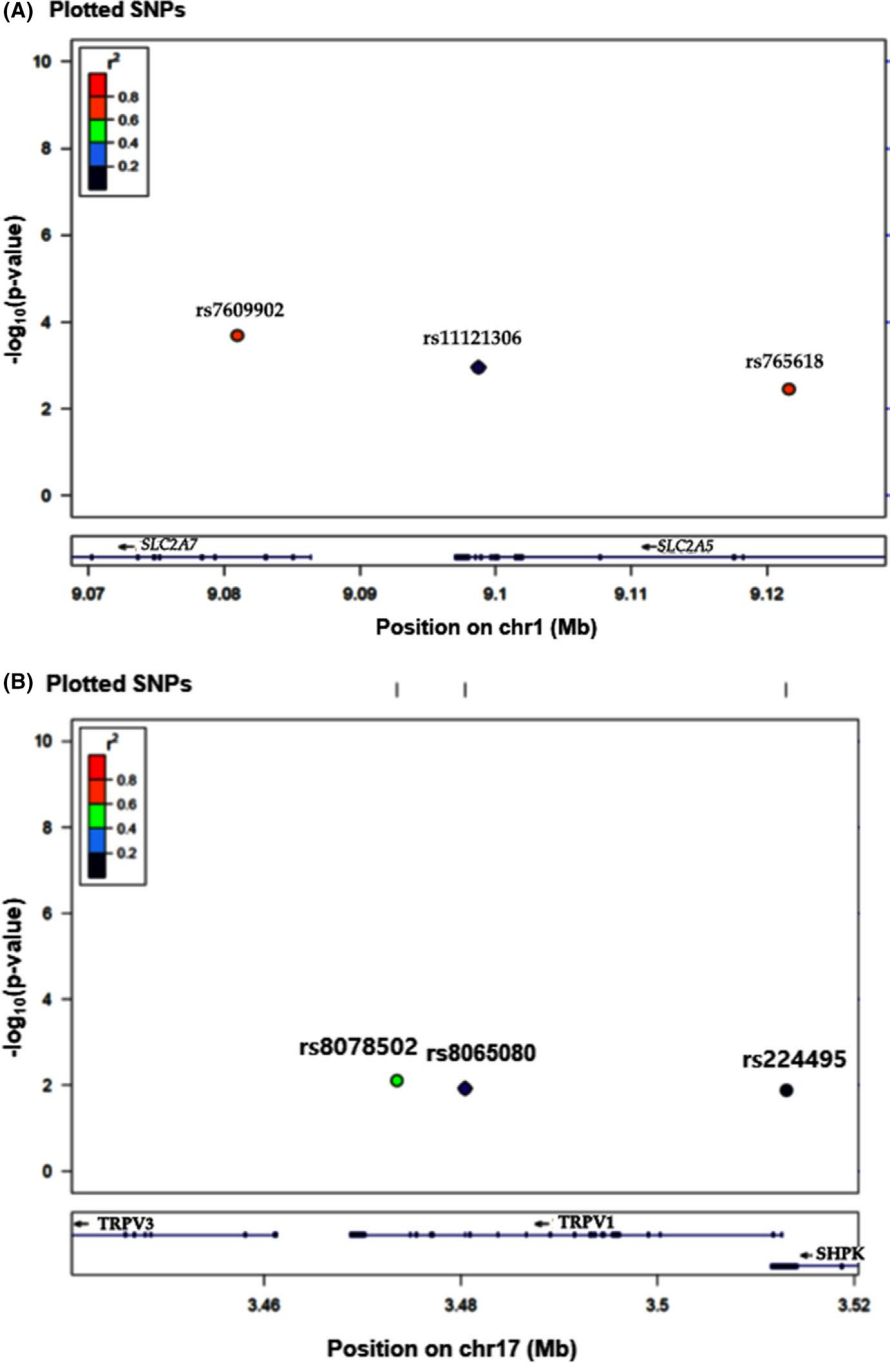
LocusZoom plot for analysis of local linkage disequilibrium (LD). (a) SNPs in chromosome 1. (b) SNPs in chromosome 17

We found 8 SNPs in the taste receptor‐related genes that have a significant association with sweet taste perception after adjusting for age, gender, residence area, and BMI at the significance level of .05 (Table 1). The 8 SNPs were satisfied for HWE, and their MAF was 0.1–0.35. *TRPV1_*rs8065080 was located in config reference with missense, *TAS1R2*_rs61761364 and *TRPV1*_rs224495 were located near the gene 5’ area and the remaining 5 SNPs were in the intron area. *SLC2A7_*rs7699022 and *TRPM5*_rs1965606 had a significant association with type 2 diabetes (Table 1). These genes included in the model were not conserved in chromosome 1 (Figure 1a) and 17 (Figure 1b; LD block with D’ <0.8).

**TABLE 1:** Characteristics of genetic variants that affect sweet taste preference

CHR	GENE	SNP	Location	Mi/Ma	OR (CI) for sweet‐ taste preference	*p*‐value^1^	OR (CI) for glucose intolerance	*p*‐value^2^	MAF	HWE	Functional consequence [References]
1	*TAS1R2*	rs61761364	19164603	A/G	0.900 (0.814–0.996)	.0482	0.975 (0.877–1.085)	.646	0.102	0.862	near‐gene‐3 [NM_152232.2]
1	*SLC2A5*	rs11121306	9098742	A/G	1.146 (1.056–1.244)	.0011	1.359 (0.881–1.043)	.332	0.182	0.069	Intron variants [NM_003039.1]
1	*SLC2A7*	rs769902	9080966	C/T	1.152 (1.068–1.243)	.0002	0.909 (0.840–0.983)	.017	0.218	0.575	Intron [NM_207420.2]
1	*SLC2A5*	rs765618	9121623	T/C	1.138 (1.043–1.240)	.0035	0.963 (0.881–1.053)	.407	0.156	0.808	Intron [NM_003039.1]
11	*TRPM5*	rs1965606	2632553	C/G	.905 (0.827–0.989)	.0289	1.123 (1.023–1.232)	.014	0.135	0.750	Intron [NM_181798.1]
17	*TRPV1*	rs224495	3513127	A/G	1.099 (1.020–1.185)	.0132	1.033 (0.956–1.116)	.410	0.218	0.418	near‐gene‐5 [NM_080704.3]
17	*TRPV1*	rs8065080	3480447	T/C	1.087 (1.019–1.160)	.0119	0.956 (0.894–1.023)	.194	0.349	0.888	Missense [NM_080706.3]
17	*TRPV1*	rs8078502	3473534	G/C	1.105 (1.027–1.189)	.0076	0.969 (0.898–1.045)	.409	0.238	0.500	Intron [NM_080705.3]

Note

Chr, chromosome; CI, 95% confidence intervals; *GLUT*, glucose transporter; HWE, Hardy–Weinberg equilibrium; Ma, major alleles; MAF, minor allele frequency; Mi, minor alleles; OR, odds ratio; *SLC*, solute carrier family; *TAS1R2*, taste receptor type 1 member 2; *TRPM5*, transient receptor potential cation channel subfamily M member 5; *TRPV1*, transient receptor potential cation channel subfamily V member 1.

Significance of odds ratio (OR) and confidence intervals (CI) for sweet state^1^ or glucose intolerance^2^ after adjusting for age, gender, residence area, and body mass index.

### 3.2 Baseline characteristics of the subjects according to GRS status

GRS was calculated by summing the number of sweet taste preference alleles of 8 genetic variants. Age, BMI, waist circumference, and gender were not different among the GRS groups. Serum glucose and insulin levels at fasting states, hemoglobin A1c, area under the curve of glucose, and insulin during OGTT, HOMA‐IR, and HOMA‐B were not different among the GRS groups after adjusting for age, gender, residence area, and BMI (Table S1). Smoking and drinking status, physical activity, and mental stress did not differ among the GRS groups.

Daily energy intake was not significant according to the GRS groups after adjusting for age, gender, residence area, and BMI (Table 2). Carbohydrates, protein, and fat intake based on energy intake were not significant according to the GRS groups after adjusting for age, gender, residence area, and BMI (Table S1). As expected, sweetness preference was much higher in the high GRS group than the low GRS group (*p* < .0001; Table 3). The intake of sucrose‐containing foods especially soda and coffee with sucrose was higher in the high GRS group than the low GRS group (Table 2). Food intake rich in sucrose and sweet food intake was higher in the high GRS than the low GRS (Table 2). However, the intake of other foods was not significantly different among the GRS groups (Table 2).

**TABLE 2:** Food intake according to genetic risk scores (GRS)

	Low‐GRS (*n* = 2,176)	Medium‐GRS (*n* = 3,574)	high GRS (*n* = 2,754)	*p*‐value^3^
Daily energy intake (kcal/day)	1924 ± 687	1910 ± 715	1939 ± 746	.3490
Sweetness preference (%)	23.7	31.5	44.8	<.0001
Units (servings/week)				
Sucrose‐rich food intake^1^	20.6 ± 0.3^b^	21.4 ± 0.3^ab^	21.7 ± 0.3^a^	.0279
Sweet‐food intake^2^	31.7 ± 0.4^b^	32.4 ± 0.4^ab^	33.0 ± 0.4^a^	.0466
Fruits	29.0 ± 0.3	28.8 ± 0.3	28.3 ± 0.2	.0954
Soda	4.0 ± 0.1^b^	4.2 ± 0.1^ab^	4.3 ± 0.1^a^	.0432
Cake	2.1 ± 0.0	2.1 ± 0.0	2.1 ± 0.0	.7071
Pastry	2.8 ± 0.1	2.7 ± 0.1	2.8 ± 0.1	.5521
Snack	3.3 ± 0.1	3.3 ± 0.1	3.4 ± 0.1	.8415
Milk	18.2 ± 0.4	17.9 ± 0.4	18.1 ± 0.3	.0660
Yogurt	13.8 ± 0.3	13.4 ± 0.3	13.4 ± 0.3	.6865
Ice cream	3.3 ± 0.1	3.4 ± 0.1	3.4 ± 0.0	.4298
Coffee	10.8 ± 0.1	10.9 ± 0.1	11.0 ± 0.1	.6258
Coffee with sugar	9.4 ± 0.1	9.6 ± 0.1	9.8 ± 0.1	.1075
Chocolate/candy	8.1 ± 0.3	8.1 ± 0.2	8.1 ± 0.2	.9931

Note

GRS was calculated by summing the allele with sweet taste preference of 8 selected SNPs (*TAS1R2*_rs61761364, *SLC2A5*_rs11121306, *SLC2A7*_rs769902, *SLC2A5*_rs765618, *TRPM5*_rs1965606, *TRPV1*_rs224495, *TRPV1*_rs8065080, and *TRPV1*_rs8078502).

GRS was divided into 3 groups by tertiles; 1–4, low GRS; 5–7, Medium‐GRS; >7, high GRS.

^1^ Intake of summing the servings of coffee with sugar, soda including coke, and candy per week.

^2^ Intake of summing the servings of coffee with sugar, soda including coke, candy, cake, snack, pastry, and ice cream per week.

^3^ Significant difference among the groups after adjusting for age, sex, residence area, BMI, daily energy intake, income, education levels, physical activity, alcohol intake, smoking status, HOMA‐IR, stress levels, and sugar, fat, and carbohydrate intake.

^a,b^ Means without a common letter differ in the same row at *p* < .05.

**TABLE 3:** Adjusted odds ratios and 95% confidence intervals of genetic risk scores (GRS) for taste perception and sugar intake

	Model 1			Model 2	
	Low GRS^1^	Medium GRS	High GRS	Medium GRS	High GRS
Sweet taste	1	1.141 (0.998–1.304)	1.282 (1.130–1.454)***	1.162 (1.011–1.336)	1.298 (1.137–1.481)***
Sour taste	1	1.071 (0.937–1.226)	1.034 (0.911–1.174)	1.094 (0.954–1.254)	1.046 (0.919–1.190)
Salty taste	1	1.102 (0.960–1.264)	0.983 (0.864–1.118)	1.116 (0.970–1.285)	0.980 (0.859–1.118)
Oily taste	1	1.118 (0.976–1.280)	1.081 (0.952–1.228)	1.120 (0.975–1.287)	1.085 (0.951–1.237)
Spicy taste	1	1.049 (0.910–1.210)	0.987 (0.864–1.128)	1.054 (0.911–1.219)	0.985 (0.859–1.130)
Sucrose‐rich food intake^2^	1	1.187 (1.016–1.386)	1.251 (1.080–1.448)**	1.182 (1.003–1.392)	1.254 (1.075–1.463)*
Sweet‐food intake^3^	1	1.149 (0.984–1.341)	1.203 (1.039–1.392)*	1.192 (1.004–1.415)	1.224 (1.042–1.439)*

Note

GRS was calculated by summing the allele with sweet taste preference of 8 selected SNPs (*TAS1R2*_rs61761364, *SLC2A5*_rs11121306, *SLC2A7*_rs769902, *SLC2A5*_rs765618, *TRPM5*_rs1965606, *TRPV1*_rs224495, *TRPV1*_rs8065080, and *TRPV1*_rs8078502).

GRS was divided into 3 groups by tertiles; 1–4, low GRS. 5–7, medium GRS, >7, high GRS.

Model 1; Adjusted for age, sex, residence area, BMI, daily energy intake, income, education levels. Model 2: model 1 plus physical activity, alcohol intake, smoking status, HOMA‐IR, stress levels, and sugar, fat, and carbohydrate intake.

^1^ The low GRS group was used as a reference group.

^2^ Frequencies of summing the servings of sucrose‐containing foods including coffee with sugar, soda (Coke), and candy per week.

^3^ Frequencies of summing the servings of sucrose‐containing foods including coffee with sugar, soda (Coke), candy, cake, pastry, and ice cream per week.

* Significantly different from the low GRS group at *p* < .05, ** *p* < .01, *** *p* < .001.

### 3.3 Association of GRS with taste preference and sucrose‐containing food intake

As expected, GRS was positively associated with sweet taste perception in model 1 and model 2 according to parameters of the adjustment (*p* < .0001; Table 3). GRS did not have an association with other tastes such as sour, salty, spicy, and oily taste (Table 4). ORs for sweet taste were higher in the high GRS group than the low GRS group after adjusting for age, gender, residence area, BMI, and income and education level (model 1; OR = 1.282; CI = 1.011–1.336) and adjusting for parameters in model 1 plus physical activity, alcohol intake, smoking status, HOMA‐IR, fat and carbohydrate intake, stress level and sucrose intake (model 2; OR = 1.298 CI = 1.137–1.481) (Table 3). Consistent with sweet taste preference, sucrose intake had a higher ORs in the high GRS group than in the low GRS group in model 1 and model 2 (Table 3).

**TABLE 4:** Adjusted odds ratios and 95% confidence intervals of metabolic syndrome and its components according to genetic risk scores (GRS) of sweet taste‐related genes

	Model 1			Model 2	
	Low GRS^1^ (*n* = 2,176)	Medium GRS (*n* = 3,574)	High GRS (*n* = 2,754)	Medium GRS (*n* = 3,574)	High GRS (*n* = 2,754)
Metabolic syndrome	1	1.032 (0.861–1.237)	1.049 (0.884–1.245)	0.991 (0.820–1.198)	1.009 (0.844–1.207)
Glucose intolerance	1	0.903 (0.788–1.035)	0.861 (0.757–0.979)*	0.898 (0.780–1.034)	0.887 (0.777–1.013)
Waist	1	1.006 (0.824–1.228)	1.095 (0.908–1.320)	1.015 (0.820–1.256)	1.118 (0.916–1.364)
Blood pressure	1	0.971 (0.847–1.113)	0.940 (0.826–1.069)	0.990 (0.854–1.148)	0.950 (0.827–1.093)
HDL	1	0.939 (0.807–1.094)	0.979 (0.848–1.130)	0.939 (0.800–1.102)	0.968 (0.833–1.126)
Serum triglyceride	1	0.897 (0.861–1.132)	0.964 (0.848–1.097)	1.013 (0.879–1.167)	0.989 (0.866–1.130)
HOMA‐IR	1	0.911 (0.799–1.039)	0.944 (0.869–1.068)	0.919 (0.801–1.054)	0.974 (0.855–1.108)
HOMA‐B	1	1.010 (0.879–1.160)	0.981 (0.861–1.118)	1.038 (0.897–1.201)	1.001 (0.871–1.149)

Note

GRS was calculated by summing the allele with sweet taste preference of 8 selected SNPs (*TAS1R2*_rs61761364, *SLC2A5*_rs11121306, *SLC2A7*_rs769902, *SLC2A5*_rs765618, *TRPM5*_rs1965606, *TRPV1*_rs224495, *TRPV1*_rs8065080, and *TRPV1*_rs8078502).

GRS was divided into 3 groups by tertiles; 1–4, low GRS; 5–7, Medium‐GRS; >7, high GRS.

Model 1; Adjusted for age, sex, residence area, BMI, daily energy intake, income, education levels. Model 2: model 1 plus physical activity, alcohol intake, smoking status, HOMA‐IR, stress levels, and sugar, fat, and carbohydrate intake.

^1^ The low GRS group was used as a reference group.

* Significantly different from the low GRS group at *p* < .05.

### 3.4 Association of GRS with parameters related to metabolic syndrome

The GRS was not associated with the risk of metabolic syndrome (Table 4). In individual components of metabolic syndrome, ORs for glucose intolerance were lower in the high GRS than the low GRS (reference) only in model 1 (Table 4). However, waist circumference, blood pressure and serum HDL cholesterol, and triglyceride levels were not associated with GRS. The risk of HOMA‐IR and HOMA‐B did not have associations with GRS (Table 4).

### 3.5 Interactions between GRS and lifestyles to increase sucrose intake and glucose intolerance

Adding sucrose is reported to be positively associated with BMI only in women (Deglaire et al., 2015). Although there was no significant interaction of GRS with gender to affect sugary food intake and waist circumferences, ORs for sucrose‐containing food intake were higher in the high GRS than those for the low GRS only in women, not men (Table 5). Thus, women were more impacted by GRS for sucrose‐containing food intake. However, ORs for glucose intolerance were not significantly different between low GRS and high GRS in both men and women.

**TABLE 5:** Interaction of genetic risk score (GRS) with lifestyles to affect the sweet taste

	Sucrose‐rich food intake			Glucose intolerance	
	Low GRS^1^ (*n* = 2,176)	Medium GRS (*n* = 3,574)	High GRS (*n* = 2,754)	Medium GRS (*n* = 3,574)	High GRS (*n* = 2,754)
Gender interaction (*p*‐value)	.236^1^			.948^2^	
Men	1	1.175 (0.973–1.419)	1.234 (0.998–1.538)	0.889 (0.731–1.082)	0.861 (0.717–1.035)
Women	1	0.969 (0.792–1.187)	1.279 (1.016–1.610)*	0.896 (0.741–1.083)	0.849 (0.709–1.017)
Mental stress interaction (*p*‐value)	.048			.781	
Low stress	1	1.090 (0.916–1.296)	1.304 (1.076–1.581)*	0.893 (0.756–1.055)	0.833 (0.712–0.975)*
High stress^3^	1	1.018 (0.806–1.284)	1.187 (0.920–1.532)	0.892 (0.703–1.133)	0.896 (0.717–1.121)
Alcohol intake interaction (*p*‐value)	.213			.396	
Low alcohol	1	1.047 (0.904–1.212)	1.272 (1.078–1.501)*	0.870 (0.748–1.013)	0.858 (0.744–0.991)*
High alcohol^4^	1	1.063 (0.762–1.483)	1.078 (0.768–1.515)	1.067 (0.776–1.467)	0.879 (0.665–1.179)
Smoking interaction (*p*‐value)	.252			.880	
Nonsmoker	1	1.008 (0.836–1.216)	1.369 (1.107–1.693)**	0.870 (0.728–1.039)	0.827 (0.699–0.979)*
Smokers^5^	1	1.160 (0.941–1.429)	1.146 (0.915–1.436)	0.905 (0.733–1.117)	0.880 (0.722–1.073)
Physical activity interaction (*p*‐value)	.531			.028	
Low physical activity	1	1.081 (0.894–1.306)	1.169 (0.952–1.436)	0.801 (0.666–0.964)*	0.886 (0.745–1.054)
High physical activity^6^	1	1.010 (0.825–1.235)	1.386 (1.103–1.740)**	1.001 (0.822–1.220)	0.808 (0.670–0.975)**

Note

GRS was calculated by summing the allele with sweet taste preference of 8 selected SNPs (*TAS1R2*_rs61761364, *SLC2A5*_rs11121306, *SLC2A7*_rs769902, *SLC2A5*_rs765618, *TRPM5*_rs1965606, *TRPV1*_rs224495, *TRPV1*_rs8065080, and *TRPV1*_rs8078502).

GRS was divided into 3 groups by tertiles; 1–4, low GRS; 5–7, Medium‐GRS; >7, high GRS.

*p*‐value for interactions between GRS and lifestyles for sugar intake^1^ and waist circumferences^2^ after adjusting confounding such as age, sex, residence area, BMI, daily energy intake, income, education levels, physical activity, alcohol intake, smoking status, HOMA‐IR, stress levels, and sugar, fat, and carbohydrate intake.

High mental stress^3^, high alcohol intake^4^, and high physical activity^6^ were defined as ≥7 mental stress scores, ≥20 g alcohol/day, and ≥10 physical activity scores, respectively. ^5^Nonsmoking included never‐smoking and past‐smoking.

* Significantly different from the low GRS group at *p* < .05, ** *p* < .01.

**FIGURE 2:**
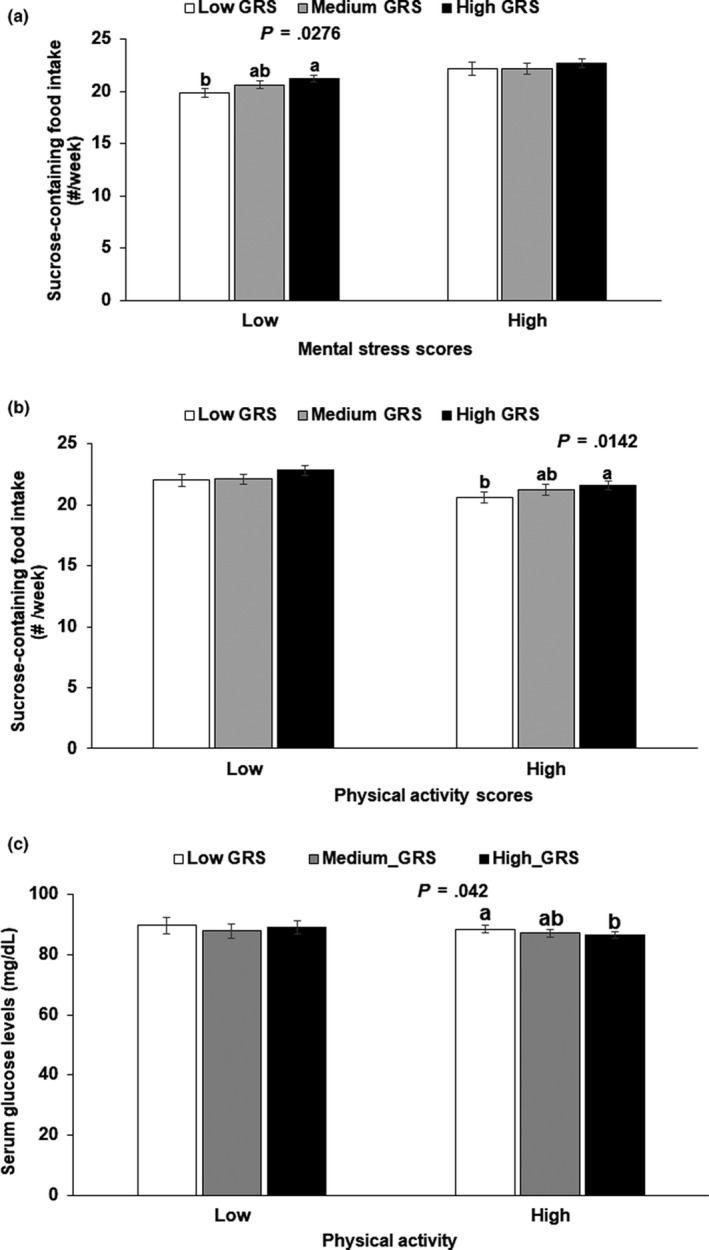
Sucrose intake and waist circumference based on GRS according to low and high mental stress and physical activity in Ansan/Ansung cohort. (a) Sucrose‐containing food intake of the GRS groups according to mental stress. (b) Sucrose‐containing food intake of the GRS groups according to physical activity. (c) Serum glucose levels of the GRS groups according to physical activity. Low and high mental stress states were < 10 and ≥ 10 of metal stress scores, respectively. GRS was calculated by a summation of the number of risk alleles from 8 selected SNPs and three groups were made by 0–4 (low GRS), 5–7 (Medium‐GRS), and >7 (high GRS) according to GRS scores. White, gray, and black bars indicated carriers with the low‐, medium‐, and high GRS group, respectively. Bars represent means ± standard error. ^a,b^Different letters indicate significant differences among the GRS groups

Mental stress had an interaction with GRS to affect sucrose‐containing food intake after adjusting for age, gender, residence area, BMI, daily energy intake, alcohol intake, smoking status, income, education level, fat, and carbohydrate intake (*p* = .048; Table 5). Subjects with low mental stress consumed significantly more sucrose with increasing GRS. Only in the low mental stress group, frequencies of sucrose‐containing food intake were higher in the high GRS group than the low GRS group (*p* = .0276, Figure 2a). It indicated that in the high stress state, sucrose‐rich food intake increased in all subjects regardless of genetic impact. GRS did not interact with glucose intolerance (Table 5).

There was no interaction of GRS with alcohol intake in sucrose intake and glucose intolerance (Table 5). However, ORs for sucrose intake was higher in high GRS than low GRS only in low alcohol intake but not in the high intake. Glucose intolerance was lower in the high GRS than the low GRS only in low alcohol intake (Table 5).

The smoking status also did not have any interaction of GRS with sucrose‐containing food intake and glucose intolerance (Table 5). However, OR for sucrose intake was higher in the high GRS than in low GRS and that for glucose intolerance was lower in the high GRS than the low GRS only in nonsmokers.

There was an interaction of GRS with physical activity to influence glucose intolerance, but no sucrose intake (Table 5). In the high physical activity situation, sucrose intake increased by 1.39‐fold in the high GRS than the low GRS (Table 5). Sucrose intake was higher in subjects of the high GRS than those of the low GRS only in high physical activity (Figure 2b). Serum glucose levels were lower in the high GRS than the low GRS in high physical activity (*p* = .042; Figure 2c).

## 4 DISCUSSION

The genetic variants of taste receptors and their signaling affect sweet taste preference and they may modulate sweet taste signaling pathways differently to influence metabolic changes. We examined the hypothesis that genetic variants that increase sweet taste preference would cause the carriers to consume more sucrose‐containing foods and alter energy, lipid, and glucose metabolism and that they would have an interaction with lifestyles. We found that subjects with high GRS of *TAS1R2*_rs61761364, *SLC2A5*_rs11121306, *SLC2A7*_rs769902, *SLC2A5*_rs765618, *TRPM5*_rs1965606, *TRPV1*_rs224495, *TRPV1*_rs8065080, and *TRPV1*_rs8078502 had higher sweet taste preference. Subjects with high GRS for sweet taste preference had more sucrose‐containing foods. However, they had less glucose intolerance. Subjects with the high GRS increased sucrose‐containing food intake by 1.3‐fold only in low mental stress and those with the high GRS lowered glucose intolerance by 0.808‐fold in high physical activity.

Taste or gustatory perception is the sensation produced by the substances to stimulate taste receptors. The signals from the taste receptors are delivered into the brain and the activation in the brain modulates the peripheral metabolism. The genetic variants of taste receptors that affect sweet taste preferences influence sucrose‐containing food intake and possibly other food groups like alcohol intake and smoking. Sweetness perception is initiated in a dimer of the *TAS1R2* and *TAS1R3* receptor proteins in the taste buds of the tongue (Inoue et al., 2004). The *TAS1R3* gene is involved in the transduction of sweet taste by the response to not only sucrose but also saccharin and δ‐phenylalanine. Its genetic variants affect the number of sweet foods people with those variants consume (Inoue et al., 2004). The allelic changes of *TAS1R3* modify sweet taste recognition and sucrose‐containing food intake: Obese people with the G allele of rs12033832 (G > A) had a higher threshold for sweet taste causing them to consume more sucrose, but lean people with the G allele showed the opposite trends (Dias et al., 2015). Moreover, the response to sucrose in people carrying the *TAS1R3* genetic variants is higher at relatively lower sucrose dosages (120 mM) than a higher dosage (300 mM) (Rawal, Hayes, Wallace, Bartoshuk, & Duffy, 2013). This indicates that over 300 mM sucrose may be the ceiling, and at higher amounts, the receptors would not detect any differences in sweet state with different genetic variants. The present study demonstrated that *TAS1R2*_rs61761364 had a significant association with sweet taste preference, but the subjects with its minor allele did not have significantly different intakes of sucrose‐containing foods.

Sweet taste perception is modulated by not only *TAS1R* but also other taste receptors and taste receptor signaling mediators such as cluster of differentiation 36 (*CD36*) and glucose transporters (Mizuta et al., 2008; Pioltine et al., 2016). The present study showed that genetic variants of glucose transporters such as *SLC2A5* and *SLC2A7* were associated with sweet taste preference. *TRPM5*, a cation channel of the *TRP* superfamily, is co‐expressed with taste receptors *TAS1Rs* and *TAS2Rs* and acts as taste receptor signal transducers (Ishimaru & Matsunami, 2009). *TRPM5* is also known to play a crucial role in sweet taste perception (Talavera et al., 2005). *TRPM5* is related to high‐temperature sensitivity and increasing temperatures between 15°C and 35°C markedly stimulate the gustatory nerve response to sweet compounds to enhance sweet taste perception (Talavera et al., 2005). The present study also showed that *TRPM5*_rs1965606 had a significant association with sweet taste preference. Although *TRPV1*, another member of the *TRP* family, is reported to recognize the pungency of capsaicin, it is also involved in salt and sour taste. However, *TRPV1* may be involved in sweet taste sensation since taste perceptions for different tastes are influenced by each other. The genes involved in different taste perceptions are interrelated to modulate food intake (Loper, La Sala, Dotson, & Steinle, 2015). In the present study, *TRPV1*_rs224495, rs8065080, and rs8078502 had significant associations with sweet taste preference.

Sweet taste receptors are expressed in the oral and in extraoral tissues such as the gut, pancreatic islets, brain, bladder, bone, and adipose tissues (Laffitte, Neiers, & Briand, 2014). The receptors transmit information about sucrose and glucose contents of ingested food into the related organs to prepare for metabolizing glucose (Laffitte et al., 2014). The activation of *TAS1R3* by glucose intake suppresses to ghrelin release in ghrelin‐producing cells in the stomach (Laffitte et al., 2014). Glucose intake is involved in regulating appetite in part by taste receptors. *TAS1R2* and *TAS1R3* in enteroendocrine cells in the small intestine secrete glucagon‐like peptide and glucose‐dependent insulinotropic peptide to regulate glucose homeostasis (Jang et al., 2007). In addition to *TAS1R*, the deletion of α‐gustducin, a mediator of the *TAS1R* signaling pathway, does not increase the secretion of glucagon‐like peptide‐1 after direct glucose administration. The knockout of either *TAS1R3* or α‐gustducin in mice suppresses the increase of sodium–glucose cotransporter‐1 (*SGLT‐1*) expression after oral carbohydrate loading (Jang et al., 2007). Glucose activates the cell surface glucose‐sensing receptor *TAS1R3* and promotes its metabolism especially in pancreatic β cells (Kojima, Medina, & Nakagawa, 2017). TRPV1 rs161364 and rs8065080 have a negative association with glucose intolerance (Song, Paik, Park, & Song, 2016), consistent with the present study. The present study showed that high GRS has a negative association with glucose intolerance indicating that subjects with high GRS had a greater preference for sweet taste and better glucose utilization. Consistent with the results, subjects with type 2 diabetes have a lower preference for sweet taste than nondiabetic subjects (Yu et al., 2014), suggesting that subjects with high GRS had better glucose metabolism.

Sweet taste preference is related to not only the genetic background but also environmental factors including physical activity, psychological constructs, and mental stress. Both conditions are linked to changes in taste preference. Mental and physical stress increase serum cortisol levels that affect sweet taste recognition. The intensity and duration of bitter, sour, and sweet taste are reduced by mental stress when compared to those before the stressors within the same session (Al'Absi, Nakajima, Hooker, Wittmers, & Cragin, 2012). This indicates that higher sucrose concentration is needed to recognize sweet taste during stress. In the present study, the genetic impact was shown only in low mental stress since the reduction of sweet taste in high mental stress mitigated the genetic impact of taste preference. Glucose intolerance showed a lower association with high GRS only in the high physical activity subgroup.

The strength of this study was that it is the first study to show that genetic variants related to sweet taste preference were associated with the consumption of more sucrose‐containing foods and affected glucose metabolism and waist circumferences, and they had an interaction with mental stress. The genes linked to taste receptor signaling, *TAS1R2*, *SLC2A5*, *SLC2A7*, *TRPM5*, and *TRPV1* were involved in sweet taste preference. However, this study had some limitations. First, the results could not identify cause and effect relationships since this was a cross‐sectional study in a large cohort. Second, self‐reported taste preference, dietary, and lifestyle questionnaires are subject to error since the standard for each parameter is different in each subject. Third, a few selected SNPs were studied but the selected genes were previously reported to link to taste preference. Finally, no replication study has been conducted since no data are available for taste perception in other Korea cohort studies including City and Rural cohort.

## 5 CONCLUSION

In conclusion, a high GRS of 8 SNPs from *TAS1R2*, *SLC2A5*, *SLC2A7*, *TRPM5*, and *TRPV1* had a positive association with sweet taste preference, compared to low GRS. Subjects with genetically sweet taste preference consumed more sucrose‐containing foods. However, the subjects with the high GRS who had the sweetness preference had higher glucose tolerance. Mental stress and physical activity interacted with genetic sweet taste preference and sucrose intake. Genetic impacts on sweet taste preference and sucrose intake existed in the situation of low mental stress and high physical activity. In low mental stress, the participants with high GRS increased sucrose‐containing food intake more than those with low GRS. Meanwhile, in high physical activity, the participants with the high GRS improved glucose tolerance, compared to the low GRS.

## CONFLICT OF INTEREST

No conflict of interest.

## ETHICAL APPROVAL

The study was approved by the institutional review board of the Korean National Institute of Health for the KoGES and Hoseo University. Written informed consent was received from each participant.

## Supporting information

Table S1

## References

[fsn31632-bib-0001] Ahn, Y. , Kwon, E. , Shim, J. E. , Park, M. K. , Joo, Y. , Kimm, K. , … Kim, D. H. (2007). Validation and reproducibility of food frequency questionnaire for Korean genome epidemiologic study. Europran Journal of Clinical Nutrition, 61, 1435–1441. 10.1038/sj.ejcn.1602657 17299477

[fsn31632-bib-0002] Al'Absi, M. , Nakajima, M. , Hooker, S. , Wittmers, L. , & Cragin, T. (2012). Exposure to acute stress is associated with attenuated sweet taste. Psychophysiology, 49, 96–103. 10.1111/j.1469-8986.2011.01289.x 22091733 PMC3240721

[fsn31632-bib-0003] Allen, A. L. , McGeary, J. E. , & Hayes, J. E. (2014). Polymorphisms in TRPV1 and TAS2Rs associate with sensations from sampled ethanol. Alcoholism, Clinical and Experimental Research, 38, 2550–2560. 10.1111/acer.12527 PMC4211991 25257701

[fsn31632-bib-0004] Blednov, Y. A. , & Harris, R. A. (2009). Deletion of vanilloid receptor (TRPV1) in mice alters behavioral effects of ethanol. Neuropharmacology, 56, 814–820. 10.1016/j.neuropharm.2009.01.007 19705551 PMC2775500

[fsn31632-bib-0005] Chan, C. B. , Hashemi, Z. , & Subhan, F. B. (2017). The impact of low and no‐caloric sweeteners on glucose absorption, incretin secretion, and glucose tolerance. Applied Physiology, Nutrition, and Metabolism, 42, 793–801. 10.1139/apnm-2016-0705 28407474

[fsn31632-bib-0006] Deglaire, A. , Mejean, C. , Castetbon, K. , Kesse‐Guyot, E. , Hercberg, S. , & Schlich, P. (2015). Associations between weight status and liking scores for sweet, salt and fat according to the gender in adults (The Nutrinet‐Sante study). European Journal of Clinical Nutrition, 69, 40–46. 25074389 10.1038/ejcn.2014.139

[fsn31632-bib-0007] Dias, A. G. , Eny, K. M. , Cockburn, M. , Chiu, W. , Nielsen, D. E. , Duizer, L. , & El‐Sohemy, A. (2015). Variation in the TAS1R2 gene, sweet taste perception and intake of sugars. Journal of Nutrigenetics and Nutrigenomics, 8, 81–90. 26279452 10.1159/000430886

[fsn31632-bib-0008] Hong, K. W. , & Oh, B. (2012). Recapitulation of genome‐wide association studies on body mass index in the Korean population. International Journal of Obesity, 2005(36), 1127–1130. 10.1038/ijo.2011.202 22041983

[fsn31632-bib-0009] Hwang, J.‐Y. , Lee, H. J. , Go, M. J. , Jang, H. B. , Park, S. I. , Kim, B.‐J. , & Lee, H.‐J. (2016). An integrative study identifies KCNC2 as a novel predisposing factor for childhood obesity and the risk of diabetes in the Korean population. Scientific Reports, 6, 33043 10.1038/srep33043 27623749 PMC5022012

[fsn31632-bib-0010] Inoue, M. , Reed, D. R. , Li, X. , Tordoff, M. G. , Beauchamp, G. K. , & Bachmanov, A. A. (2004). Allelic variation of the Tas1r3 taste receptor gene selectively affects behavioral and neural taste responses to sweeteners in the F(2) hybrids between C57BL/6ByJ and 129P3/J mice. The Journal of Neuroscience, 24, 2296–2303. 10.1523/JNEUROSCI.4439-03.2004 14999080 PMC1400603

[fsn31632-bib-0011] Ishimaru, Y. , & Matsunami, H. (2009). Transient receptor potential (TRP) channels and taste sensation. Journal of Dental Research, 88, 212–218. 10.1177/0022034508330212 19329452 PMC2876190

[fsn31632-bib-0012] Jang, H.‐J. , Kokrashvili, Z. , Theodorakis, M. J. , Carlson, O. D. , Kim, B.‐J. , Zhou, J. , … Egan, J. M. (2007). Gut‐expressed gustducin and taste receptors regulate secretion of glucagon‐like peptide‐1. Proceedings of the National Academy of Sciences, 104(38), 15069–15074. 10.1073/pnas.0706890104 PMC1986614 17724330

[fsn31632-bib-0013] Kim, D. S. , Kim, B. C. , Daily, J. W. , & Park, S. (2018). High genetic risk scores for impaired insulin secretory capacity doubles the risk for type 2 diabetes in Asians and is exacerbated by Western‐type diets. Diabetes/Metabolism Research and Reviews, 34(1), e2944. 10.1002/dmrr.2944 29048714

[fsn31632-bib-0014] Kobayashi, Y. , Habara, M. , Ikezazki, H. , Chen, R. , Naito, Y. , & Toko, K. (2010). Advanced taste sensors based on artificial lipids with global selectivity to basic taste qualities and high correlation to sensory scores. Sensors, 10, 3411–3443. 10.3390/s100403411 22319306 PMC3274227

[fsn31632-bib-0015] Kojima, I. , Medina, J. , & Nakagawa, Y. (2017). Role of the glucose‐sensing receptor in insulin secretion. Diabetes, Obesity & Metabolism, 19(Suppl 1), 54–62. 10.1111/dom.13013 28880472

[fsn31632-bib-0016] Laffitte, A. , Neiers, F. , & Briand, L. (2014). Functional roles of the sweet taste receptor in oral and extraoral tissues. Current Opinion in Clinical Nutrition and Metabolic Care, 17(4), 379–385. 10.1097/MCO.0000000000000058 24763065 PMC4059820

[fsn31632-bib-0017] Lee, A. A. , & Owyang, C. (2017). Sugars, sweet taste receptors, and brain responses. Nutrients, 9, 653 10.3390/nu9070653 PMC5537773 28672790

[fsn31632-bib-0018] Loper, H. B. , La Sala, M. , Dotson, C. , & Steinle, N. (2015). Taste perception, associated hormonal modulation, and nutrient intake. Nutrition Reviews, 73, 83–91. 10.1093/nutrit/nuu009 PMC4555787 26024495

[fsn31632-bib-0019] Matsushita, Y. , Mizoue, T. , Takahashi, Y. , Isogawa, A. , Kato, M. , Inoue, M. , … Tsugane, S. (2009). Taste preferences and body weight change in Japanese adults: The JPHC Study. International Journal Obesity, 33, 1191–1197. 10.1038/ijo.2009.153 19636316

[fsn31632-bib-0020] Merigo, F. , Benati, D. , Cristofoletti, M. , Osculati, F. , & Sbarbati, A. (2011). Glucose transporters are expressed in taste receptor cells. Journal of Anatomy, 219, 243–252. 10.1111/j.1469-7580.2011.01385.x 21592100 PMC3162243

[fsn31632-bib-0021] Mizuta, E. , Kokubo, Y. , Yamanaka, I. , Miyamoto, Y. , Okayama, A. , Yoshimasa, Y. , … Morisaki, T. (2008). Leptin gene and leptin receptor gene polymorphisms are associated with sweet preference and obesity. Hypertension Research, 31, 1069–1077. 10.1291/hypres.31.1069 18716353

[fsn31632-bib-0022] Murovets, V. O. , Bachmanov, A. A. , & Zolotarev, V. A. (2015). Impaired glucose metabolism in mice lacking the Tas1r3 taste receptor gene. PLoS ONE, 10, e0130997 10.1371/journal.pone.0130997 26107521 PMC4479554

[fsn31632-bib-0023] Paik, H. Y. (2008). Dietary reference intakes for Koreans (KDRIs). Asia Pacific Journal of Clinical Nutrition, 17(Suppl 2), 416–419. 18460441

[fsn31632-bib-0024] Park, S. , Ham, J. O. , & Lee, B. K. (2015). Effects of total vitamin A, vitamin C, and fruit intake on risk for metabolic syndrome in Korean women and men. Nutrition, 31, 111–118. 10.1016/j.nut.2014.05.011 25466654

[fsn31632-bib-0025] Pearlman, M. , Obert, J. , & Casey, L. (2017). The association between artificial sweeteners and obesity. Current Gastroenterology Reports, 19, 64 10.1007/s11894-017-0602-9 29159583

[fsn31632-bib-0026] Pioltine, M. B. , de Melo, M. E. , Santos, A. , Machado, A. D. , Fernandes, A. E. , Fujiwara, C. T. , … Mancini, M. C. (2016). Genetic variation in CD36 is associated with decreased fat and sugar intake in obese children and adolescents. Journal of Nutrigenetics and Nutrigenomics, 9, 300–305. 28237985 10.1159/000455915

[fsn31632-bib-0027] Pippitt, K. , Li, M. , & Gurgle, H. E. (2016). Diabetes mellitus: Screening and diagnosis. American Family Physicians, 93, 103–109. 26926406

[fsn31632-bib-0028] Rabbee, N. , & Speed, T. P. (2006). A genotype calling algorithm for affymetrix SNP arrays. Bioinformatics, 22, 7–12. 10.1093/bioinformatics/bti741 16267090

[fsn31632-bib-0029] Rawal, S. , Hayes, J. E. , Wallace, M. R. , Bartoshuk, L. M. , & Duffy, V. B. (2013). Do polymorphisms in the TAS1R1 gene contribute to broader differences in human taste intensity? Chemical Senses, 38, 719–728. 10.1093/chemse/bjt040 24000232 PMC3777563

[fsn31632-bib-0030] Robino, A. , Bevilacqua, L. , Pirastu, N. , Situlin, R. , Di Lenarda, R. , Gasparini, P. , & Navarra, C. O. (2015). Polymorphisms in sweet taste genes (TAS1R2 and GLUT2), sweet liking, and dental caries prevalence in an adult Italian population. Genes & Nutrition, 10, 485 10.1007/s12263-015-0485-z 26268603 PMC4534477

[fsn31632-bib-0031] Rother, K. I. , Conway, E. M. , & Sylvetsky, A. C. (2018). How non‐nutritive sweeteners influence hormones and health. Trends in Endocrinology and Metabolism, 29, 455–467. 10.1016/j.tem.2018.04.010 29859661

[fsn31632-bib-0032] Schlesinger, S. , Chan, D. S. M. , Vingeliene, S. , Vieira, A. R. , Abar, L. , Polemiti, E. , … Norat, T. (2017). Carbohydrates, glycemic index, glycemic load, and breast cancer risk: A systematic review and dose‐response meta‐analysis of prospective studies. Nutrition Reviews, 75, 420–441. 10.1093/nutrit/nux010 28969357

[fsn31632-bib-0033] Shin, Y. , Lee, S. , & Kim, Y. (2018). Sweet preference associated with the risk of hypercholesterolemia among middle‐aged women in Korea. Journal of Atherosclerosis and Thrombosis, 25, 1215–1221. 10.5551/jat.43000 29618672 PMC6249356

[fsn31632-bib-0034] Song, S. , Paik, H. Y. , Park, M. , & Song, Y. (2016). Dyslipidemia patterns are differentially associated with dietary factors. Clinical Nutrition, 35, 885–891. 10.1016/j.clnu.2015.06.002 26140958

[fsn31632-bib-0035] Suez, J. , Korem, T. , Zeevi, D. , Zilberman‐Schapira, G. , Thaiss, C. A. , Maza, O. , … Elinav, E. (2014). Artificial sweeteners induce glucose intolerance by altering the gut microbiota. Nature, 514, 181–186. 10.1038/nature13793 25231862

[fsn31632-bib-0036] Talavera, K. , Yasumatsu, K. , Voets, T. , Droogmans, G. , Shigemura, N. , Ninomiya, Y. , … Nilius, B. (2005). Heat activation of TRPM5 underlies thermal sensitivity of sweet taste. Nature, 438, 1022–1025. 10.1038/nature04248 16355226

[fsn31632-bib-0037] Yamagishi, K. , & Iso, H. (2017). The criteria for metabolic syndrome and the national health screening and education system in Japan. Epidemiology and Health, 39, e2017003 10.4178/epih.e2017003 28092931 PMC5343105

[fsn31632-bib-0038] Yu, J. H. , Shin, M.‐S. , Lee, J. R. , Choi, J. H. , Koh, E. H. , Lee, W. J. , … Kim, M.‐S. (2014). Decreased sucrose preference in patients with type 2 diabetes mellitus. Diabetes Research and Clinical Practice, 104, 214–219. 10.1016/j.diabres.2014.02.007 24629412

