# Supplementary material for: Mental stress and physical activity interact with the genetic risk scores of the genetic variants related to sweetness preference in high sucrose‐containing food and glucose tolerance
Source: Food Sci Nutr. 2020 May 21;8(7):3492–503. doi: 10.1002/fsn3.1632 (PMC7382188; doi:10.1002/fsn3.1632)
Supplement: Supplementary file 1 — Table S1 [file FSN3-8-3492-s001.docx]

Supplemental Table 1. Baseline characteristics of subjects according to GRSs that affect sweet-taste preference

|  | Low-GRS  (n=2,176) | Medium-GRS (n=3,574) | High-GRS (n=2,754) | P value^1^ |
| --- | --- | --- | --- | --- |
| Age (years) | 52.2±9.1 | 52.0±8.9 | 52.2±8.8 | 0.4084 |
| Gender (male %) | 1,070 (25.6) | 1,749 (41.8) | 1,364 (32.6) | 0.9404 |
| BMI (kg/m^2^) | 24.7±3.0 | 24.6±3.0 | 24.6±3.2 | 0.5933 |
| Waist circumference (cm) | 82.6±8.8 | 82.3±8.7 | 82.8±8.9 | 0.0639 |
| Fasting serum glucose (mg/dL) | 89.0±22.8 | 87.5±21.4 | 87.9±21.7 | 0.1102 |
| Fasting serum insulin (μIU/mL) | 7.70±4.32 | 7.49±4.58 | 7.64±5.26 | 0.2590 |
| Area under the curve of serum glucose | 873±277 | 860±276 | 862±263 | 0.2275 |
| Area under the curve of insulin | 166±131 | 165±135 | 168±138 | 0.6718 |
| Hemoglobin A1c (%) | 5.83±0.95 | 5.80±0.96 | 5.80±0.93 | 0.2923 |
| HOMA-IR | 1.71±1.55 | 1.63±1.07 | 1.68±1.28 | 0.0902 |
| HOMA-B | 150.3±137.6 | 155.0±155.7 | 152.6±145.5 | 0.5272 |
| Smoking (yes, %) | 25.3 | 25.7 | 25.5 | 0.9887 |
| Drinking (No, %) | 58.6 | 58.9 | 58.9 | 0.3586 |
| Regular exercise (Yes, %) | 50.7 | 53.1 | 51.8 | 0.2032 |
| Mental stress^2^ (High, %) | 12.3 | 12.6 | 13.2 | 0.5532 |
| Carbohydrate (En %) | 70.8±7.0 | 70.8±6.8 | 70.7±7.1 | 0.4776 |
| Protein (En%) | 13.6±2.5 | 13.6±2.3 | 13.6±2.5 | 0.5024 |
| Fat (En%) | 14.4±5.4 | 14.4±5.3 | 14.4±5.5 | 0.5823 |

Genetic risk scores (GRS) was calculated by summing the allele with sweet-taste preference of 8 selected SNPs (*TAS1R2*_rs61761364, *SLC2A5*_rs11121306, *SLC2A7*_rs769902, *SLC2A5*_rs765618, *TRPM5*_rs1965606, *TRPV1*_rs224495, *TRPV1*_rs8065080, and *TRPV1*_rs8078502). GRS was divided into 3 groups by tertiles; 1-4, Low-GRS; 5-7, Medium-GRS; >7, High-GRS. HOMA-IR, homeostasis model assessment for insulin resistance; HOMA-B, homeostasis model assessment for insulin secretion.

^1^Significant difference after adjusting for age, gender, residence area and BMI.

^2^High mental stress defined as ≥7 mental stress scores.
